# Chitosan Nanoformulations of Mycosporine-like Amino Acid (MAA)-Rich Extracts from *Mazzaella laminarioides* Effectively Protect Human Keratinocytes Against UVA Radiation Damage

**DOI:** 10.3390/ijms262110394

**Published:** 2025-10-25

**Authors:** Osmán Vásquez, Braulio Contreras-Trigo, Eileen Castillo, Neriel Contreras, Jessica Lemus, Felipe A. Zuniga, Karina Oyarce, Dariela Núñez, Víctor Díaz-García, Patricio Oyarzún

**Affiliations:** 1 Facultad de Ingeniería, Universidad San Sebastián, Concepción 4080871, Chile; ovasquezv@correo.uss.cl (O.V.); bcontrerast@docente.uss.cl (B.C.-T.); neriel.contreras@gmail.com (N.C.); jlemusj@docente.uss.cl (J.L.); 2 Facultad Ciencias, Universidad San Sebastián, Concepción 4080871, Chile; ceileenv@docente.uss.cl (E.C.); karina.oyarce@gmail.com (K.O.); 3 Departamento de Bioquímica Clínica e Inmunología, Facultad de Farmacia, Universidad de Concepción, Concepción 4070386, Chile; fzuniga@udec.cl; 4 Facultad de Ciencias, Universidad Católica de la Santísima Concepción, Concepción 4090541, Chile; dnunez@ucsc.cl

**Keywords:** chitosan, nanoparticle, red algae, MAAs, photoprotection, UVA

## Abstract

Mycosporine-like amino acids (MAAs) are secondary metabolites of interest for the development of natural sunscreens, owing to their antioxidant activity and ultraviolet radiation (UVR)-absorbing properties. MAA-rich aqueous extracts obtained from the Chilean red alga *Mazzaella laminarioides* (locally known as luga cuchara) were analyzed by HPLC and loaded into chitosan nanoparticles (CSNPs), with an encapsulation efficiency of 90.1%. The resulting CS nanoformulations (CSNFs) were characterized by FTIR spectroscopy, DLS and TEM microscopy, confirming the presence of nanoparticles with a core diameter of 94 ± 11 nm and FTIR absorption bands accounting for CS functional groups. Pre-treatment of HaCaT keratinocytes with CSNFs conferred complete protection against low-to-moderate UVA doses (5, 10, 15, and 30 J/cm^2^). Remarkably, cells still retained a protection efficacy of 64.7% under lethal UVA exposure (60 J/cm^2^), with gene expression evidence suggesting the activation of a compensatory stress response to photo-oxidative damage. CSNFs were also capable of restoring cell viability in post-treatment experiments at UVA doses of 30 J/cm^2^ (100% cell viability) and 60 J/cm^2^ (~43% cell viability). This is the first demonstration that nanoencapsulation of an MAA-rich algal extract yields superior UVA photoprotection in human keratinocytes compared with non-encapsulated MAA-based formulations, contributing to the effort of developing eco-friendly sunscreens.

## 1. Introduction

Over the past few decades, significant depletion of the stratospheric ozone layer has raised concerns about the effects of increased solar ultraviolet radiation (UVR), which is classified into three spectral ranges: UVA (400–315 nm), UVB (315–280 nm), and UVC (280–100 nm) spectral ranges. Low-energy UVA accounts for ~95% of the UVR that reaches the Earth’s surface, which can penetrate deeply into human skin and induces photo-oxidative damage to DNA and other cellular components through over-generation of reactive oxygen species (ROS) [1]. Consequently, prolonged exposure to this component of solar UVR has been linked to premature skin aging and carcinogenesis [2,3,4]. Furthermore, epidemiological evidence suggests that UVA range plays a central role in the development and progression of cutaneous melanoma, which is the most lethal form of skin cancer and one of the fastest-growing cancers worldwide [5,6].

The use of sunscreen is paramount to protect the epidermis against sun-induced damage, which typically relies on a combination of organic and inorganic filters to ensure broad-spectrum UV protection. However, a growing body of evidence suggests synthetic UVR filters may cause side-effects on human skin, including health problems associated with endocrine disruption, reproductive organs and central nervous system development [7]. In addition, the European Chemicals Agency (ECHA) and the Environmental Effects Assessment Panel (EEAP) of the United Nations Environment Programme (UNEP) have raised concerns about the bioaccumulation and adverse ecotoxicological effects of these compounds on fragile marine ecosystems [8,9,10], including coral bleaching [11] and hormonal disruption in fish [12,13].

In this context, naturally occurring UV-absorbing compounds from botanical and marine sources are gaining increasing attention as photoprotective constituents in sunscreen formulations [14,15]. In particular, marine algae have evolved effective mechanisms to protect themselves from UVR-induced adverse effects, with mycosporine-like amino acids (MAAs) playing a central role in this defense strategy [16,17]. MAAs are a family of small UVR-absorbing intracellular compounds (<400 Da) biosynthesized via a branch of the shikimic acid pathway in organisms that live in environments with high sunlight exposure. The aromatic structures of these metabolites confer unique photochemical and photophysical properties, being considered among the most potent natural UVA-absorbing compounds due to their exceptionally high molar extinction coefficients (ε = 12,400–58,800 M^−1^ cm^−1^) [18]. These compounds also inhibit the formation of UV-induced reactive photoproducts thanks to their strong antioxidant properties [18,19], exhibiting additional desirable attributes such as photostability [20], anti-photoaging [21] and anti-inflammatory properties [22]. In addition, the photoprotective properties of MAAs have been demonstrated in skin cell lines [23,24] and in mouse models following topical application [19,25,26].

On the other hand, sustainable biopolymer-based nanoparticles are being explored by virtue of their bioactive properties and excellent controlled-release performance for natural active ingredients [27,28]. Among these, chitosan nanoparticles (CSNPs) have demonstrated high efficacy as dermal drug delivery carriers for biomedical and cosmetic applications [29], owing to their biocompatibility with human tissues [30] and their remarkable antioxidant, antiviral, antimicrobial and anti-inflammatory properties [31]. A previous study highlighted the unique combination of physicochemical and biological properties exhibited by CS-MAAs biofilms to support the development of UV-absorbing green materials [32]. However, only a few studies have explored the nanodelivery of algal extracts and algae-derived bioactive compounds using CS as a carrier to improve their release properties. In particular, Maghraby et al. (2022) encapsulated an extract from a red alga (*Jania rubens*) in CSNPs and characterized the release profile of antioxidant compounds, underscoring the advantages of encapsulation for applications where sustained release is desirable [33]. More recently, Abdullah Abdel-Aal et al. (2025) reported nanoencapsulation of bioactive metabolites from a brown alga (*Turbinaria triquetra*) and demonstrated that chitosan nanoparticles (from now on referred to as “CSNPs”) conferred greater therapeutic efficacy in rats relative to the free extract [34]. Likewise, CSNPs have been successfully assessed to improve stability and bioactivity of algae-derived phlorotannins (from *Sargassum ilicifolium*) [35,36] and metabolites with biomedical applications obtained from other botanical sources (e.g., phenolic acids [37,38] and resveratrol [39]).

This work reports, for the first time, the use of nanochitosan to encapsulate and evaluate an MAA-rich aqueous algal extract for UVA photoprotection. *Mazzaella laminarioides* (locally known as luga cuchara), an edible and economically important red macroalga widely distributed along the Chilean coast, was employed as the source material to prepare the extract and to analyze its MAA composition. The resulting CS nanoformulations (from now on referred to as “CSNFs”) were comprehensively characterized (FTIR, TEM, DLS, XRD and TGA) and tested in vitro in terms of their ability to mitigate or suppress UVA-induced damage in human keratinocytes.

## 2. Results

### 2.1. Extraction and Analysis of the MAA Content

**Figure 1:**
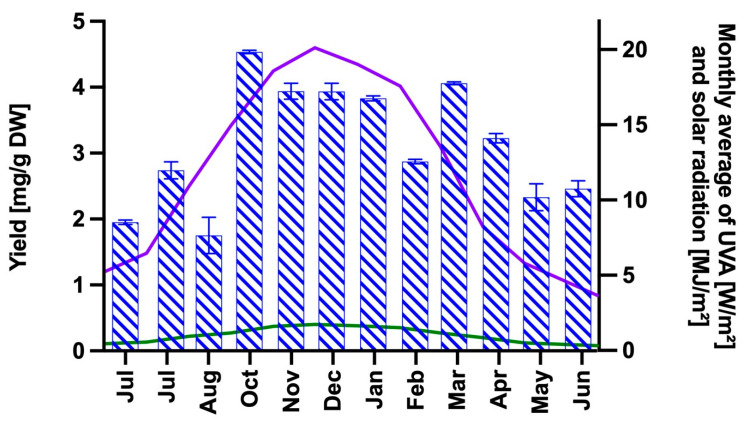
MAAs extraction yield obtained between July 2021 and June 2022, expressed in mg of total MAAs per g dry weight (DW) of alga (blue dashed columns, left axis). The lines represent the monthly averages of total solar radiation (purple; right axis) and UVA radiation (green; right axis) measured over the same time period in the coastal zone of the Biobío region. Solar radiation data were measured by the meteorological station of the Chilean Institute of Agricultural Research (INIA), while surface UVA data are estimated by the NASA CERES and accessed via the POWER Climatology API, Version: v2.5.15 (NASA, 2025).

Aqueous extracts enriched in mycosporine-like amino acids (MAAs) were prepared from *M. laminarioides* collected and analyzed monthly at Caleta Tumbes from July 2021 to June 2022 (Figure 1). Total MAA content ranged from 2–5 mg g^−1^ DW, with a maximum of 4.54 mg g^−1^ DW observed in October.

We identified four MAAs in the aqueous extracts of the alga samples (shinorine, mycosporine-glycine, palythine and asterin-330), based on their characteristic absorption maxima and HPLC retention times (Figure S3). Minor peaks occasionally appearing with absorption in the UVA region were not considered in order to be consistent with previous works [40]. The MAA porphyra-334 was tentatively confirmed in our study by HPLC techniques, consistent with the widespread occurrence of this compound in red algae (Rhodophyta) [41]. Thus, spiking the algal extract with a porphyra-334 internal standard produced an apparent increase in the area of the principal HPLC peak (t_R_ = 1.884 min) (Figure S4). Additional assays of chromatographic co-elution lend further support to the presence of both porphyra-334 (Figure S5) and shinorine (Figure S6) in the algal extract. In particular, the elution time of the porphyra-334 standard (t_R_ = 1.742 min) closely matched that of the peak presumed to represent this compound in the chromatograms (t_R_ 1.7 min). Moreover, the corresponding area of this peak increased from 537 (algal extract) to 859 and 891 in the samples spiked with the porphyra-334 standard (duplicates).

### 2.2. Characterization of the Aqueous MAA-Rich Extracts

The purification of the aqueous algal extract was carried out by gel filtration using a resin with a fractionation range of 0–700 Da, allowing the simultaneous elution of MAAs (~200–400 Da) and phenolic compounds of low molecular weights (TPC was 0.92 GAE/g DW). The elution profiles of MAAs (Figure S7) and total phenols (Figure S8) confirm the co-elution of both type of compounds between fractions #4 (4 mL) and #20 (20 mL). The highest concentrations of MAAs (82.2 mg/L) and phenolic compounds (97.98 mg/L) were obtained upon passing 10 mL and 11 mL of the mobile phase, respectively.

The ORAC analysis of the extract (from algae collected in October) revealed a strong antioxidant capacity of 1.76 µM Trolox equivalents (TE), which was slightly lower than that of ascorbic acid (2.27 µM TE) and comparable to the MAAs standards of shinorine (2.22 µM TE), porphyra-334 (1.16 µM TE) and mycosporine-glycine (1.28 µM TE). This result confirms the capability of the extract to quench free radicals and to expedite the removal of UVA-induced reactive oxygen species (ROS).

### 2.3. Nanoencapsulation of the MAA-Rich Extracts in Chitosan

**Figure 2:**
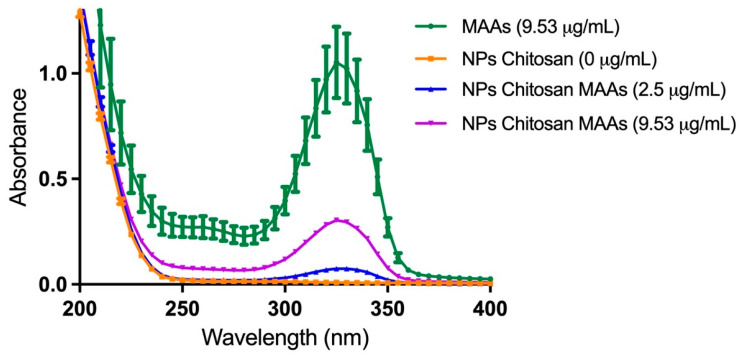
UV-Vis absorption spectra of MAA_PE_ (green), free CSNPs (orange), and CSNPs loaded with 2.5 μg/mL MAAs (blue) and 9.53 μg/mL MAAs (purple).

The ionotropic gelation method achieved an encapsulation efficiency of 90.1%, as determined from the mass balance between the initial MAA content (111 µg) and the non-encapsulated fraction (11 µg). In addition, the loading capacity reached a high value of 90.9%. Figure 2 presents the UV-Vis spectrograms of the MAA-rich extract of *M. laminariodes* and the CSNFs, highlighting three main spectral regions: (i) a chitosan-related absorption band (200–250 nm); (ii) a phenols-related absorption region (250–290 nm) and (iii) the MAAs-distinctive absorption region (290–360 nm). The absorption maximum at ~330 nm accounts for a substantial fraction of shinorine and porphyra-334, which constitute a substantial portion of the total MAA content in the extract. Furthermore, the CSNPs spectrogram lacks this peak due to the absence of characteristic absorption bands of chitosan in the UVB range. Figure S9 shows the critical wavelength (λc = 384.7 nm), which is defined as the wavelength below which 90% of the area under the absorbance (extinction) curve is contained. Together with the UVA/UVB ratio (1.651769), both parameters support broad-spectrum coverage with substantial UVA protection.

**Figure 3:**
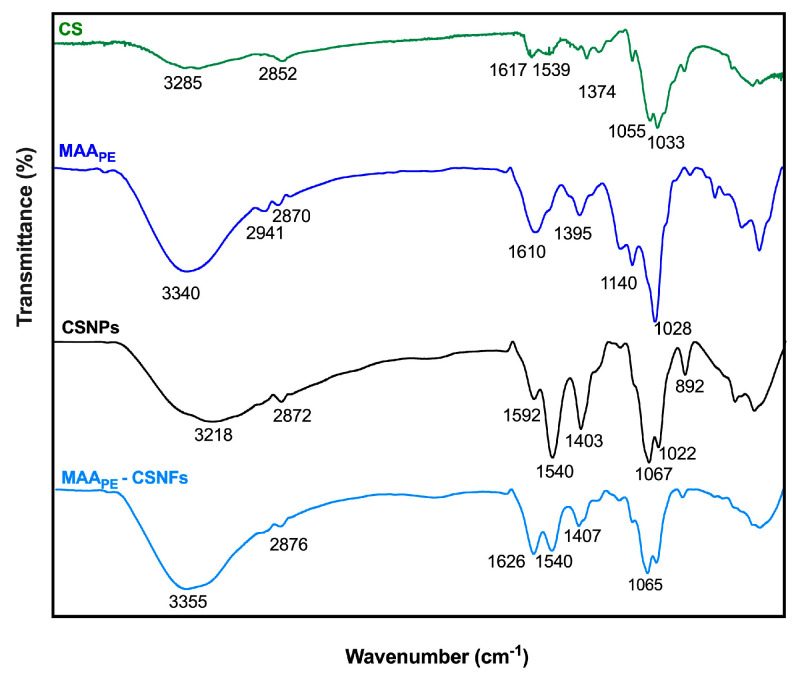
FTIR spectra of CS (green), MAA_PE_ (blue), CSNPs (black) and MAA-CSNFs (light blue).

FTIR characterization of CS, MAA_PE_, CSNPs, and MAA_PE_-CSNFs is presented in Figure 3. The spectrum of CS (green line) displays a broad peak at 3285 cm^−1^, which is attributed to the -OH and N-H stretching vibrations of primary amines. This peak becomes more pronounced in the CSNPs (black line), indicating strong hydrogen bonding. In addition, peaks at 1617 cm^−1^ and 1539 cm^−1^ in the CS spectrum correspond to C=O and N-H bending vibrations of the residual amide groups from deacetylated chitin. These peaks increase in intensity and shift to 1592 cm^−1^ and 1540 cm^−1^, respectively, due to interaction with the phosphate groups from sodium tripolyphosphate (TPP) [29]. Regarding the MAAs spectrum (blue line), a broad peak observed at 3340 cm^−1^ is attributed to -OH functional groups and N-H stretching vibrations. The peaks at 2941 cm^−1^ and 2870 cm^−1^ are associated with C-H stretching vibrations, while the bands at 1610 cm^−1^ and 1395 cm^−1^ correspond to -NH_2_ and -COOH functional groups, respectively. Finally, the spectrum of MAA-CSNFs (light blue line) reveals a characteristic peak at 1626 cm^−1^ that is specifically associated with the -NH_2_ groups of MAAs in the nanoformulation, while the broad peak at 3355 cm^−1^ reflects overlapping -OH and N-H groups from both CS and MAAs.

**Figure 4:**
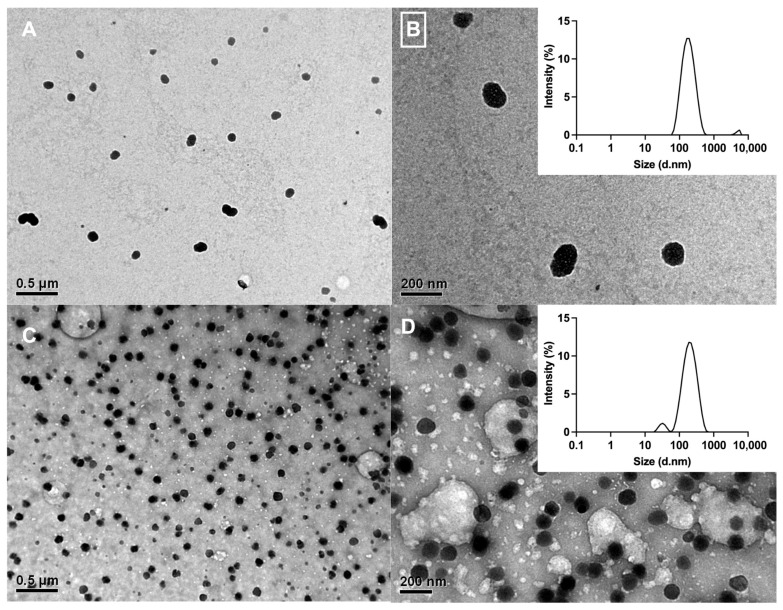
TEM micrographs of CSNPs (**A**,**B**) and MAAs-loaded CSNFs (**C**,**D**). Inset figures indicate the size distribution of empty (**B**) and MAAs-loaded (**D**) nanoformulations.

Figure 4 presents TEM images of the nanoparticles, revealing a spherical morphology with average core diameters of 95 ± 22 nm (CSNPs) and 94 ± 11 nm (MAAs-CSNPs). These results are consistent with DLS measurements, which indicated larger particle sizes for CSNFs (220 ± 20 nm) and non-encapsulated CSNPs (190 ± 19 nm), along with low polydispersity indexes (0.251 and 0.241, respectively). DLS analysis of CSNPs confirmed their positive surface charge (+19.2 mV), while MAA-loaded CSNFs exhibited a comparable value (+20.3 mV). This marginal increase is consistent with the neutral charge of MAAs and phenolic compounds present in the extract, which are not altering the surface charge of the nanoformulations significantly. In addition, the positive charge confers considerable stability to the CSNFs by promoting electrostatic repulsion between individual particles.

**Figure 5:**
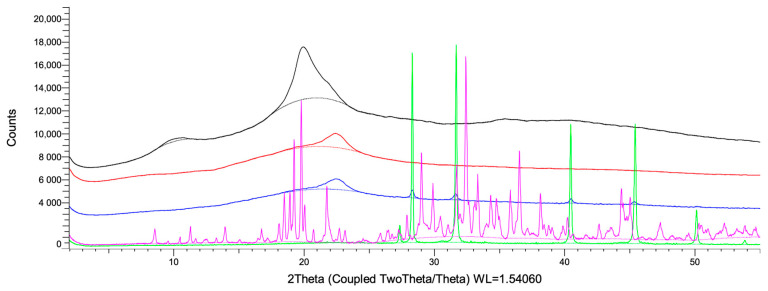
XRD patterns of pure chitosan (black), free CSNPs (red), and CSNPs loaded with MAA extract (blue), MAA extract (purple) and pure TPP (green).

XRD was used to evaluate variations in the crystalline structure of CS and the NP formulations. Figure 5 shows the typical semi-crystalline reflections of native CS at 2θ = 10.46° and 20.03° (black trace), which are absent in both empty CSNPs (red trace) and MAA-loaded CSNFs (blue trace). This change in the diffraction pattern accounts for a predominantly amorphous structure [42,43]. In contrast, CSNFs exhibited additional reflections at 2θ = 28.27°, 31.46°, 40.36°, 44.67°, and 49.54°, matching peaks characteristic of NaCl and KCl present in the MAA_RE_ (green trace; Figure S10). Finally, the XRD pattern of TPP powder displayed sharp reflections at multiple 2θ angles, confirming the crystalline nature of the crosslinking agent as documented in previous studies [44].

**Figure 6:**
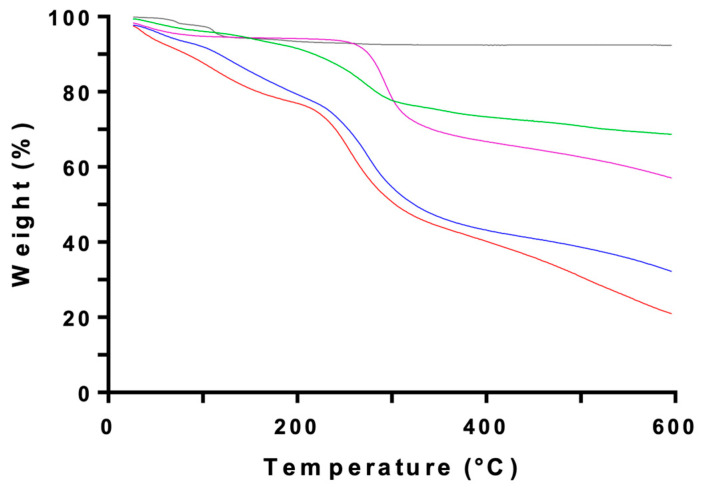
TGA profiles showing mass loss as a function of temperature for pure chitosan (purple), empty CSNPs (red), MAA-loaded CSNPs (blue), MAA extract (green), and pure TPP (black).

Figure 6 shows the TGA thermograms for the same set of samples. A slight mass loss for CS and CSNPs below and around 100 °C is attributed to the evaporation of adsorbed water [45]. CS powder exhibits its main decomposition over 229–373 °C (peak at 292 °C), with an associated mass loss of ~41% (residue ~58.7%) that is consistent with depolymerization and the decomposition of the acetyl and amine groups of chitosan [46]. CSNPs and CSNFs display a similar pattern, although the decomposition shifts to a lower temperature range (254–274 °C).

### 2.4. In Vitro Photoprotection Experiments

To investigate the protective effect of MAA-rich aqueous extracts, HaCaT cells were incubated with CSNFs either before (pre-treatment) or after (post-treatment) exposure to UVA radiation. Prior to testing photoprotection, we established that CSNPs were non-toxic to HaCaT cells at the concentrations used in this study. The experiments were conducted using environmentally and physiologically relevant UVA doses according to previous studies, including low (5–15 J/cm^2^), medium (30 J/cm^2^), and high (60 J/cm^2^) exposure levels [47,48].

#### 2.4.1. HaCaT Cells Exposure to Low-to-Medium UVA Radiation Doses

**Figure 7:**
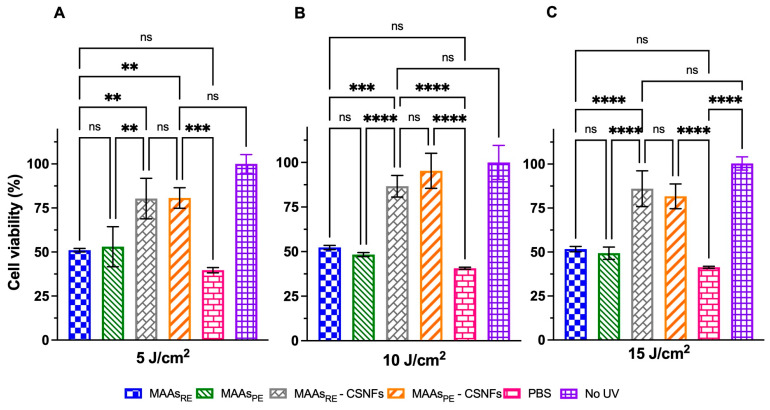
Evaluation of the photoprotective effects of free MAAs (10.2 µg/mL) and CSNFs (10.2 µg/mL MAA_RE_) administered before UVA exposure in HaCaT cells subjected to radiation doses of (**A**) 5 J/cm^2^, (**B**) 10 J/cm^2^, and (**C**) 15 J/cm^2^. Controls included UVA-unexposed cells (violet) and PBS was used as a baseline (red). Columns represent the mean ± s.d. (n = 3). Asterisks denote statistically significant differences between the treatments and controls, ** = *p* < 0.01, *** = *p* < 0.001 and **** = *p* < 0.0001. Non-significant differences are indicated as ns.

Figure 7 shows the CSNFs was highly effective in protecting keratinocytes against UVA damage, keeping ~80–95% of cell viability under the three experimental conditions (5, 10 and 15 J/cm^2^). In all cases, pre-treated HaCaT cells (grey and orange bars) maintained viability significantly above that of cells incubated with free MAAs (reduced to ~50%; blue and green bars) and regarding untreated cells (reduced to ~35–40%; red bars). As expected, non-UVA irradiated keratinocytes (purple bars) showed ~100% viability. Furthermore, no statistical differences were found when comparing raw versus semi-purified extracts.

**Figure 8:**
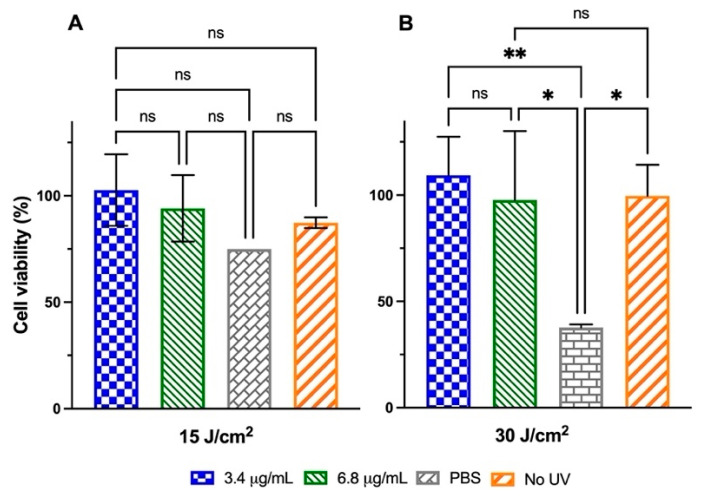
Evaluation of the photoprotective effects of CSNFs containing 3.4 µg/mL (blue) and 6.8 µg/mL (green) of MAA_RE_ in HaCaT cells subjected to UVA doses of (**A**) 15 J/cm^2^ and (**B**) 30 J/cm^2^, under post-treatment conditions. Controls included cells treated with empty CSNPs and UVA-unexposed cells. PBS was used as a baseline control. Columns represent the mean ± s.d. (n = 3). Asterisks denote statistically significant differences between the treatments and controls. * = *p* < 0.05 and ** = *p* < 0.01. Non-significant differences are indicated as ns.

The second strategy sought to evaluate whether the CSNFs exerted a protective effect on cell viability when applied to keratinocytes previously subjected to UVA irradiation (doses of 15 J/cm^2^ and 30 J/cm^2^). Notably, cell cultures incubated with CSNPs-3.4 and CSNFs-6.8 recovered up to 100% viability following exposure to 30 J/cm^2^ of UVA (Figure 8). By contrast, HaCaT cells that did not receive post-treatment only reached 38% (30 J/cm^2^) and 70% (15 J/cm^2^) viability in the assays.

#### 2.4.2. HaCaT Cells Exposure to High UVA Radiation Doses

**Figure 9:**
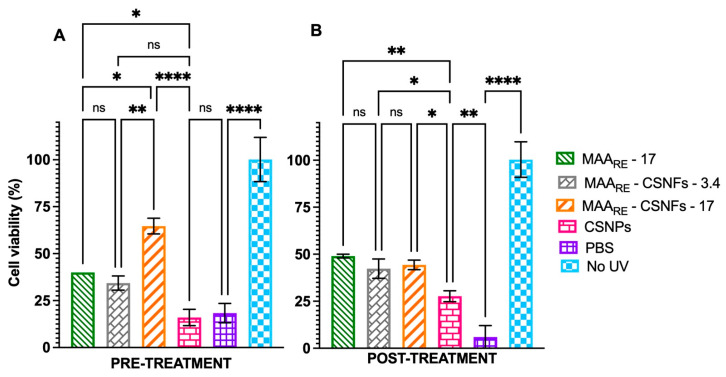
Evaluation of the photoprotective effect of free MMAs (17 μg/mL MAAs) and CSNFs (3.4 and 17 μg/mL MAA_RE_) in HaCaT cells subjected to an UVA radiation dose of 60 J/cm^2^, under (**A**) pre-treatment and (**B**) post-treatment conditions. Controls included cells treated with empty CSNPs and UVA-unexposed cells. PBS was used as a baseline control. Columns represent the mean ± s.d. (n = 3). Asterisks denote statistically significant differences between the treatments and controls. * = *p* < 0.05, ** = *p* < 0.01 and **** = *p* < 0.0001. Non-significant differences are indicated as ns.

The effectiveness of CSNFs was further investigated under drastic conditions of UVA irradiation, by exposing the HaCaT cells to 3 mW/cm^2^ during 5.6 h (60 J/cm^2^). Figure 9 confirms the lethal effect of this high UVA dose, as indicated by the reduction in viability to 18.3% (pre-treatment) and 6% (post-treatment) in untreated control cells. Interestingly, even with a high level of UVA exposure, the photoprotective effect in keratinocytes was noticeable when treated with CSNFs. Thus, CSNPs-17 provided strong protection to keratinocytes when administered before UVA exposure, resulting in 64.7% cell viability. This effect was significantly greater (*p* < 0.01) than that observed with CSNFs-3.4 (34.3% viability), additionally outperforming (*p* < 0.05) the protection offered by free MAAs to the cells at the same concentration (40% viability). However, no significant differences were observed in post-treatment experiments between the photoprotective effects delivered by CSNFs and free MAA_RE_, with cell viabilities fluctuating between ~35–45%. Furthermore, MAAs-free CSNPs exhibited a notable protective effect in post-treatment experiments (~30% viability), although this was statistically lower than that achieved with MAA-containing formulations (*p* < 0.05).

### 2.5. Expression Analysis of Genes Associated with Photo-Oxidative Damage

To gain insight into the underlying photoprotective mechanisms, we conducted RT-PCR analyses on genomic DNA from irradiated keratinocytes, using primer pairs targeting specific regions of the Cox-1 (cyclooxygenase-1) and Keap1 (Kelch-like ECH-associated protein 1) genes that are associated with inflammatory response (Cox-1) and redox balance (Keap1). In particular, Cox-1 is a constitutively expressed enzyme that plays a central role in maintaining physiological homeostasis through the biosynthesis of prostaglandins and other mediators of inflammation. On the other hand, Keap1 acts as a cytoplasmic repressor of Nrf2 (Nuclear factor erythroid 2-related factor 2) that functions as a redox sensor that releases Nrf2 upon oxidative stress to activate the transcription of cytoprotective genes involved in the antioxidant response.

**Figure 10:**
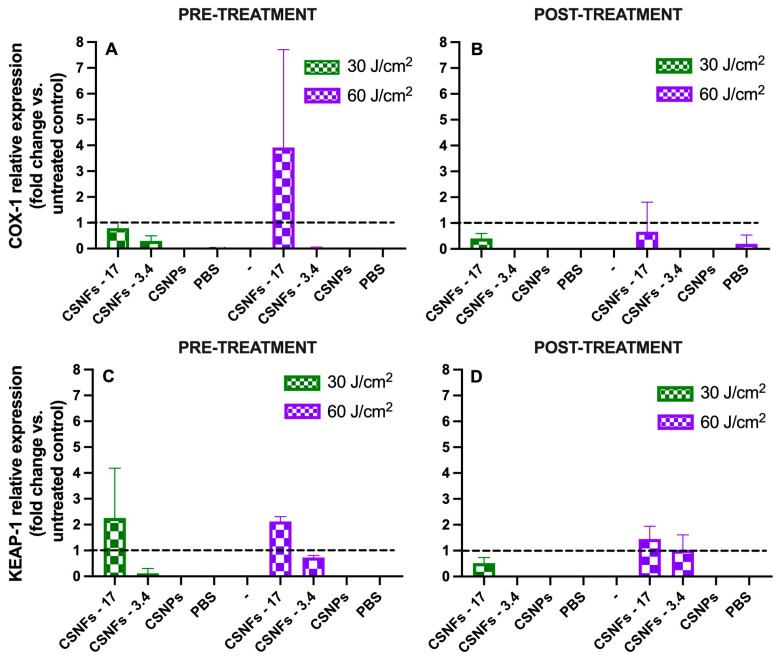
Gene expression levels of Cox-1 and Keap-1 genes in CSNFs-treated keratinocytes subjected to UVA irradiation (30 and 60 J/cm^2^). (**A**) Cox-1 expression under pre-treatment conditions; (**B**) Cox-1 expression under post-treatment conditions; (**C**) Keap-1 expression under pre-treatment conditions; (**D**) Keap-1 expression under post-treatment conditions. Treatments included CSNFs loaded with MAA_RE_ 17 µg/mL (CSNFs-17) and MAA_RE_ 3.4 µg/mL (CSNPs-3.4), as well as controls consisting of the empty CSNFs (or CSNPs) and PBS alone. Gene expression was normalized using β-actin as a housekeeping gene and all the values are expressed relative to the untreated controls (dash line; fold change = 1). Columns represent the mean ± s.d. (n = 3).

Figure 10 shows that Cox-1 and Keap1 gene expression levels were downregulated in MAAs-free controls (CSNPs and PBS) under both pre- and post-treatment conditions, in a consistent manner with observed reduction in cell viability (up to 75%) of keratinocytes subjected to a high-UVA dose (Figure 9A,B). Likewise, a reduction in Cox-1 expression was observed in keratinocytes treated with CSNFs-3.4 (at 30 J/cm^2^ UVA), suggesting this concentration of MAAs (3.4 μg/mL) was unable to induce a protective response (Figure 10A). However, cells treated with CSNFs-17 exhibited a pronounced increase on Cox-1 expression levels (2.92-fold increase) following UVA exposure (60 J/cm^2^), compared with untreated control cells (Figure 10A).

Keratinocytes subjected to UVA irradiation also showed a marked decrease in Keap1 expression in both pre- and post-treatment settings when treated with CSNPs-3.4 (Figure 10C,D). However, the cell line treated with CSNFs-17 exhibited a pronounced upregulation of Keap1, with expression levels rising more than twofold (2.25- and 2.12-fold) following UVA irradiation at 30 and 60 J/cm^2^, respectively (Figure 10C). Likewise, the treatment of previously irradiated keratinocytes (60 J/cm^2^) with CSNFs-17 also enhanced Keap1 expression by 1.45-fold compared to controls. By contrast, CSNFs-3.4 exerted minimal effects at 60 J/cm^2^, resulting in only slight changes in Keap1 expression both in pre-treatment (0.72-fold) and post-treatment (1.03-fold).

## 3. Discussion

MAAs exhibit high water solubility due to the polar nature of their cyclohexenone or cyclohexenimine ring structures. Given this, we opted to use pure water as the extraction solvent, in agreement with current trends that prioritize eco-friendly alternatives to organic solvents [49]. However, in the literature water is often combined with polar solvents such as methanol or ethanol (i.e., hydroalcoholic mixtures) to enhance extraction efficiency from algae and other botanical sources [50]. For example, previous works have investigated the photoprotective activity of algae-derived extracts on the viability of model cells exposed to UVR, reporting promising results with a methanolic extract of *Porphyra yezoensis* [51] and an ethanolic extract of *Sargassum cristaefolium* [52]. The use of algal extracts offers the potential for synergistic combinations that deliver a broad spectrum of benefits, whereas isolating individual MAAs may be counterproductive due to the loss of such complementarity. Accordingly, these extracts are of considerable interest as functional additives in cosmetic formulations [53,54], as exemplified by a commercial sunscreen formulated with liposomally encapsulated *Porphyra umbilicalis* extract [55].

The high antioxidant activity of the semi-purified extract (1.76 µM TE) is consistent with the presence of both MAAs and phenolic compounds. The latter are characterized by hydroxylated phenyl moieties that are responsible for their potent scavenging activity against free radicals and ROS. This family of compounds mainly include bromophenols, flavonoids and a variety of phenolic acids [56,57], which are well-known to provide numerous skin benefits associated with antioxidant, anti-aging, anti-wrinkle, anti-inflammatory and antimicrobial properties [58,59,60,61]. The presence of phenolic compounds additionally contributes to the broadening of the absorption peak into the UVB region of the semi-purified algal extract [62,63,64]. In particular, the appearance of mild absorbance band around λ_max_ = 280 nm is indicative of these compounds [65,66].

MAA content measured throughout one-year period fell within the previously reported range (2–5 mg g^−1^ DW) for this species [40]. Interestingly, higher yields were obtained during the warmer months (October–April) compared to the colder months (May–September), showing a seasonality pattern that is consistent with the photoprotective role of these compounds in response to solar irradiance stress (Figure 1) [67]. HPLC analysis of the extracts revealed fluctuations in the chromatographic peaks, indicating seasonal variations in the MAA content that correlated positively with higher levels of incident solar radiation. Such variations are well-described in seaweeds and depend on factors such as geographic location and environmental conditions [68].

In agreement with our findings, previous authors have also described the presence of shinorine, mycosporine-glycine, palythine and asterina-330 in the same algal species collected from the sub-Antarctic region of Chile [40,69,70]. However, discrepancies in the preliminary identification of porphyra-334 relative to other studies may occur, since MAA content in red seaweeds is highly responsive to environmental factors, such as incident light spectrum and nitrogen availability, which play critical roles in regulating their synthesis [71,72]. For example, a higher content of mycosporine-glycine has been determined in *M. laminarioides* during a period of ozone depletion in southern Chile, suggesting an adaptive response to intensified UV radiation [67].

The CSNFs were successfully developed with average diameters of 94 ± 11 nm (binding core) and 220 ± 20 nm (hydrodynamic diameter), reaching a high encapsulation efficiency (90.1%). The differences between both measurements arise because hydrodynamic diameter reported by DLS accounts not only for the nanoparticle core (TEM images), but also for the surrounding hydration layer and surface-bound coating materials formed through interactions with the medium. Furthermore, the FTIR spectra of the CSNFs align with established chitosan signatures [32]. The spectral profile of the MAA-rich extract confirms previously described chemical features of MAAs, characterized by a central ring system substituted with amino-acid residues, imino alcohols, or other modifications [73,74,75]. Similarly, the structural features of CS, CSNPs and CSNFs were confirmed by XRD and TGA, revealing changes in crystallinity and mass-loss profiles consistent with the literature. In this regard, the reduced thermal stability in the nanostructured form likely reflects decreased crystallinity caused by ionic complexation between protonated −NH_3_^+^ groups of CS and polyanionic TPP, which disrupts interchain hydrogen bonding and the native semi-crystalline packing [47]. Finally, the MAA_RE_ presents a flatter, more extended TGA profile than CS, CSNPs and CSNFs, with gradual mass losses across a broad temperature range (34–272 °C). This behavior is consistent with the heterogeneous composition of the algal extract (low-molecular-weight metabolites, inorganic salts, and bound water), which produces overlapping dehydration and decomposition events without a dominant peak.

The UVA dose employed in the photoprotection experiments fluctuated between 5 and 60 J/cm^2^, considering that a reference UVA radiation dose of 27.5 J/cm^2^ is equivalent to 1.5 h of summer sun at noon on the French Riviera (Nice) under high UV index conditions [76]. This dose is also capable of inducing minimal erythema in fair-skinned individuals [77]. UVA is indeed recognized as a strong inducer of oxidative stress, since it penetrates deeper into the human skin than UVB and triggers photoexcitation effects over endogenous photosensitizers such as flavins, melanin and porphyrins [78,79]. Although this component of solar radiation is only weakly absorbed by DNA, the resulting generation of ROS is responsible for single-strand breaks and oxidized bases that may alter DNA stability in mammalian cells [80]. Accordingly, epidemiological evidence suggests that UVA radiation contributes to the genesis of cutaneous melanoma [2,81].

Figure 7 and Figure 9 confirmed the strong photoprotective capability of CSNFs under pre-treatment conditions, by preserving cell viability near ~80–95% at low-to-medium UVA doses (5–30 J/cm^2^) and ~35–65% even at a lethal dose (60 J/cm^2^). These results further confirm previous findings by Lawrence et al. regarding the robust photoprotective effects delivered by palythine [82], which is a MAA previously identified in the *M. laminarioides* extract [40,70]. In the referenced study, palythine was tested at concentrations ranging from 3 to 100 mg/mL and demonstrated excellent efficacy in protecting keratinocytes against UVA-induced damage (at a dose of 20 J/cm^2^), underscoring the critical role of its antioxidant properties. These concentrations are comparable to those of typical UVR filters used in commercial sunscreen formulations [83]. Notably, CSNFs achieved complete photoprotection at high UVA doses using MAA concentrations orders of magnitude lower than conventional, underscoring the remarkable efficacy of the nanoencapsulation strategy. The following table benchmarks the protective efficacy of our CSNFs against comparable state-of-the-art studies, showing superior performance in preserving (pre-treatment) and restoring (post-treatment) the viability of UVA-exposed human keratinocytes (Table 1).

In pre-treatment assays, CSNF-treated keratinocytes showed a 2.3–2.6× increase in cell viability relative to untreated controls, slightly superior to previous works. However, the advantage is more pronounced in post-treatment experiments, reaching 8.5× versus 1.1× reported by Kim et al. (2014) using a hydromethanolic/non-encapsulated algal extract of laver (*Porphyra yezoensis*) to protect UVB-irradiated HaCaT keratinocytes [51]. Indeed, in post-treatment experiments, CSNFs fully restored cell viability of keratinocytes upon UVA irradiation at 15 and 30 J/cm^2^ (Figure 8). Remarkably, CSNFs were still capable of partially restoring cell viabilities up to ~43% (post-treatment assay) when increasing the UVA radiation dose to a lethal level (60 J/cm^2^) (Figure 9). However, significant differences between CSNFs and free MAAs were detected only in pre-treatment experiments (Figure 9A). The high severity of UVA-induced damage in keratinocytes subjected to post-treatment conditions was likely responsible for the lack of a measurable response to CSNPs application (Figure 9B), thereby overriding their beneficial effects [84].

It is possible to hypothesize that the enhanced photoprotective efficacy stems from nanodelivery-mediated uptake of CSNFs, by facilitating cellular internalization of MAAs and thereby reducing the concentration required for effective protection. Furthermore, the positive charge of chitosan facilitates cell attachment by promoting electrostatic interactions with negatively charged cell membranes [85]. Figure 9B likely reflects enhanced mitochondrial metabolism rather than mitogen-driven proliferation, as MTT assay measures the enzymatic reduction of tetrazolium by NAD(P)H-dependent oxidoreductases. Thus, preservation of redox balance and mitochondrial activity is expected to yield higher formazan production without requiring an increase in cell number. This interpretation is also consistent with the well-documented antioxidant and anti-inflammatory properties of CSNPs [86,87]. On the other hand, mitogenic effects in these cells (doubling time > 37 h [88]) are unlikely to manifest within the experimental window of this assay (24 h). This is also confirmed by previous reports on CSNP-based drug delivery systems, showing no cell-cycle activation and minimal cytotoxicity [89,90].

The pronounced viability recovery under lethal UVA conditions (Figure 9) is consistent with MAAs-mediated DNA repair mechanisms widely described in the literature [91]. In this regard, the Keap1-Nrf2 signaling pathway is well-known to play a crucial role in the skin’s defense against UV-induced oxidative stress, particularly to counteract the damaging effects of the UVA spectral range. Thus, Keap1 undergoes degradation upon UVA exposure, which facilitates the release and nuclear translocation of Nrf2 to initiate a cellular defense during photo-oxidative stress [92]. Interestingly, both shinorine and porphyra-334 have previously demonstrated their capability to upregulate the expression of cytoprotective genes in response to oxidative stress by activating the Nrf2 signaling pathway [10]. This mechanism relies on their capability to antagonize the cytoplasmic repressor Keap1, thereby disrupting Keap-Nrf2 binding and promoting nuclear localization of the transcription factor. Herein, we did not directly verify an in-vitro reduction in intracellular ROS accumulation. However, the antioxidant mechanisms are already well-described in studies using red algal extracts [51,93,94] and purified algal MAAs (shinorine, asterina-330, and palythine) [23]. For example, porphyra-334 has been shown to significantly reduce UVA-induced ROS levels in human skin fibroblast (CCD-986sk cells) by exerting free radical-scavenging activities [23,95].

It is worth noting that empty CSNPs yielded a significant (*p* < 0.01) restoration of viability compared with untreated control (Figure 9B). This result may be associated with cell-proliferative properties of chitosan, as this material does not absorb radiation in the UVA range. Previous studies have shown that this biopolymer exerts a stimulatory effect on cell proliferation of fibroblasts [96] and HaCaT keratinocytes [97], which is further enhanced in its nanoparticulate form due to nanoscale interactions with components of the extracellular matrix [98,99]. Furthermore, extensive literature supports the intrinsic wound-healing activity of chitosan [100,101,102], as well as its suitability as a delivery platform of natural bioactive compounds, owing to its non-toxicity, biocompatibility and biodegradability [103].

At the gene-expression level, previous studies have also provided evidence that Cox-1 expression can be modulated by specific cellular stressors and bioactive compounds [104,105]. Thus, Cox-1 downregulation in CSNFs-3.4-treated keratinocytes (at 30 J/cm^2^ UVA) suggests impaired cellular homeostasis due to a reduced basal prostaglandin-mediated cytoprotection, thereby increasing susceptibility to oxidative and inflammatory damage [106]. By contrast, the observed Cox-1 upregulation (2.92-fold increase) in keratinocytes treated with CSNFs-17, following UVA exposure (60 J/cm^2^ UVA), likely reflects an early inflammatory response to high-dose damage and may result from a compensatory activation of prostaglandin-mediated cytoprotective pathways that support tissue repair and immune regulation. We hypothesize that the increase in Keap1 expression at mRNA level represents a compensatory or early stress response, which can be followed by degradation of the Keap1 protein through post-transcriptional or post-translational mechanisms [107]. Supporting evidence comes from Palsamy et al. (2014), who showed that ROS decreases promoter DNA methylation, leading to elevated Keap1 expression and suppression of Nrf2-mediated antioxidant defenses in epithelial cells [108]. In line with this observation, Wang et al. (2020) reported that pretreatment of HaCaT keratinocytes with a phytochemical compound increased the protein levels of both Keap1 and Nrf2 [109]. Finally, Liu et al. (2011) investigated the photoprotective effects of resveratrol in human keratinocytes (HaCaT cells) exposed to UVA irradiation (0–5.592 J/cm^2^). They found a pronounced reduction in Keap1 protein levels, despite mRNA levels remaining elevated for up to 12 h post-treatment [110].

## 4. Materials and Methods

### 4.1. Biological Material

Fronds of red alga *Mazzaella laminarioides* (luga cuchara) were collected from the intertidal zone at Caleta Tumbes (36°38′24″ S 73°05′39″ W, Talcahuano, Chile), between July 2021 and June 2022. Apical portions (15 cm length) obtained from at least 50 fronds of *M. laminarioides* were transferred to plastic Petri dishes filled with filtered natural seawater and transported to the laboratory. The samples were dried at 30 °C for 72 h, then cut into small pieces of 1 cm per side and stored in the dark at room temperature (RT) for further experiments. The immortalized human keratinocyte cell line HaCaT was kindly donated by Dr. Miguel Concha at the Department of Pathology, Faculty of Medicine, Universidad Austral de Chile, Valdivia, Chile [88]. A mycosporine-serinol standard was purchased from the Laboratory of Photobiology, Central Research Services, University of Málaga (Spain), while the other MAA standards (porphyra-334, shinorina and mycosporine-glycine) were kindly donated by Dr. Kageyama, from the Meijo University, Nagoya (Japan) [111].

### 4.2. Reagents and Chemicals

HPLC-grade acetonitrile and formic acid were provided by Merck KGaA (Darmstadt, Germany). The analytical-grade reagents acetic acid, chlorophorm, isopropyl acid, ethanol and ascorbic acid were also purchased from Merck KgaA, except for sodium tripolyphosphate that was bought from Winkler (Lampa, Santiago, Chile). High glucose Dulbecco’s modified Eagle’s medium (DMEM), fetal bovine serum (FBS) and antibiotics (penicillin and streptomycin) were acquired from Corning Inc. (Corning, NY, USA). Tripsin, phosphate buffer saline (PBS) and agarose were purchased from ThermoFisher Scientific (Waltham, MA, USA). Low molecular weight chitosan (Merck, Sigma Aldrich, Saint Louis, MO, USA) with 50–190 KDa and a 75–85% degree of deacetylation, and Sephadex G-25 resin were obtained from Sigma-Merck (Darmstadt, Germany), while Macro-Prep High-S resin was purchased from Bio-Rad (Hercules, CA, USA).

### 4.3. Preparation and Analysis of Algal Extracts

MAA-rich aqueous extracts of *Mazzaella laminarioides* were prepared by hydrating 1 g of dried samples with 10 mL of nanopure water (18 MΩ of resistance) and incubated at 30 °C for 3 h. After the incubation, the total sample volume was filtered through a gauze cloth followed by filtration with 0.2 µm hydrophilic polyvinylidene fluoride (PVDF) membranes. Then, 5 mL of the MAA-rich raw extract (MAA_RE_) was stored at 4 °C and the remaining volume was subjected to a further purification step by eluting through a Sephadex G-10 column (size 1.5 × 150 cm) in nanopure water. The MAA-rich purified extract (MAA_PE_) was obtained by collecting 20 fractions of 1 mL through continuous monitoring of UV absorption in the typical MAAs range (310–360 nm) using an Epoch^TM^ Microplate Spectrophotometer (Bio-Tek Instruments, Winooski, VT, USA). Total MAA content in the algal extracts was preliminarily estimated from the absorbance at 334 nm using the shinorine extinction coefficients (ε_334_ = 44,700 M^−1^) [112]. However, more accurate quantifications for the photoprotection assays were determined by HPLC.

### 4.4. Determination of Total Phenolic Content

The total content of phenolic compounds in the algal extract was measured by Folin–Ciocalteu colorimetric assay with gallic acid used as a standard, according to Makkar 2000) [113]. Briefly, the extract (125 μL) was mixed with 62.5 μL Folin-C (1 N) and then 312.5 μL sodium carbonate (20%) solution after 2 min. The mixture was vortexed and absorbance read at 725 nm after 40 min using an Epoch^TM^ Microplate Spectrophotometer (Bio-Tek Instruments, Winooski, VT, USA). Gallic acid was used to generate the standard curve (30, 60, 90 and 120 mg/L). Total phenolic content was expressed in mg gallic acid equivalents (GAE) per g dry weight (DW) of alga.

### 4.5. HPLC Determination of the MAAs in the Algal Extract

MAAs were determined in the algal extracts by HPLC using an in-line diode array detector (Agilent series 1100, Agilent Technologies, Waldbronn, Germany) based on the retention times and absorption spectra recorded on HPLC-separated peaks between 290 and 400 nm. The mobile phase consisted of a mixture of 4% acetonitrile (*v*/*v*) and 0.1% formic acid (*v*/*v*) in water. After the extract was passed through a 0.2 μm membrane filter, sample volumes of 20 μL were injected into a C18 reversed-phase column (Zorbax Eclipse Plus C18, 150 × 4.6 mm, 5 μm pore size) and eluted isocratically at a flow rate of 1 mL/min [111]. MAAs were additionally investigated by co-chromatography with secondary MAAs standards, using the same mobile phase in an HPLC system equipped with UV detector (Agilent series 1100, Agilent Technologies, Switzerland) and a C18 reversed-phase column (Phenomenex Luna C18, 150 × 4.6 mm, 5 μm pore size). The amount of MAAs in the aqueous extract was determined by interpolating the areas of the chromatographic peaks against a standard curve of shinorine (Figure S1), which is the predominant MAA in this alga.

### 4.6. Preparation of Chitosan-TPP Nanoformulations

Encapsulation of raw (MAA_RE_) and purified (MAA_PE_) algal extracts in CSNPs was carried out by the ionotropic gelation method [114]. Briefly, the chitosan solution (1 mg/mL) was prepared by dissolving 0.1 g of chitosan in 100 mL of solution with 0.1% (*v*/*v*) of acetic acid under continuous stirring (1000 rpm) at RT, followed by pH adjustment of the mixture to 5.5 with NaOH 1 M. The aqueous extracts of MAAs were subsequently added into a beaker containing the chitosan solution to a final concentration of 10% *w*/*w*, keeping the stirring speed at 1000 rpm. The MAA-containing CSNFs were prepared with the aid of a syringe pump (Terumo, Tokyo, Japan), by dropwise addition (1 mL/h) of 1 mg/mL of TPP into the before solution (chitosan to TPP weight ratio of 9:1), under magnetic stirring (1500 rpm) at RT. The resulting CSNFs were collected from the reaction mixture by filtering through 0.22 µm polyethersulfone (PES) filters (Millipore, Merck, Darmstadt, Germany) and a final centrifugation step at 3000× *g* for 60 min to separate unincorporated MAAs, using 3 kDa Amicon ultra centrifugal filter units (Millipore Merck KGaA, Darmstadt, Germany). MAAs concentration in the eluate was determined by HPLC, as previously described.

The encapsulation efficiency (*EE*) and loading capacity (*LC*) and parameters of the nanoformulations were calculated using the following equations:

(1) $$EE(\%)=\left(\frac{{MAA}_{t}-{MAA}_{f}}{{MAA}_{t}}\right)\cdot 100$$

(2) $$LC(\%)=\left(\frac{{MAA}_{t}-{MAA}_{f}}{W}\right)\cdot 100$$

where *MAA_t_* denotes the total mass of MAAs, *MAA_f_* the mass of free (unencapsulated) MAAs quantified in the filtrate, and *W* is the dry weight of CSNFs.

### 4.7. Instrumental Characterization of CSNFs

Particle size and morphology characterization were carried out by transmission electron microscopy (TEM) with a 4 A° resolution (JEOL-JEM 1200EX-II, JEOL Instruments, Tokyo, Japan), using a Gatan CCD camera for image acquisition (Gatan Inc., Pleasanton, CA, USA). Two microliters of water suspensions of CSNPs and MAAs-CSNPs were deposited on a copper grid covered with a holey carbon film and air-dried for TEM observation. The particle size distribution and surface charge (mV) of the CSNFs were determined by dynamic light scattering (DLS) and zeta potential analyzer (Nano-ZS90, Malvern Instruments, Westboroug, MA, USA). Attenuated Total Reflectance Fourier Transform Infrared (ATR-FTIR) spectroscopy (Spectrum Two, Perkin Elmer, Waltham, MA, USA) was employed to characterize functional groups of the freeze-dried MAA extracts (freeze drier Operon FDB-5502, Operon Co., Ltd., Gimpo, Republic of Korea), CSNPs and MAAs-CSNPs. The spectra were recorded from 4000 to 400 cm^−1^, with a resolution of 4 cm^−1^ and 40 scans were co-added per spectrum. The crystalline structure of the CSNPs was analyzed by X-ray diffraction with a Bruker D4 Endeavor Diffractometer (Bruker AXS GmbH, Karlsruhe, Germany), employing CuKα radiation (λ = 1.54060 Å). The analysis was conducted at room temperature with a step angle of 0.02°, covering a 2θ range from 2° to 55°. The thermal stability of CSNFs was studied by thermogravimetric analysis (TGA) in a TGA Q5 apparatus (Waters/TA instruments, New Castle, DE, USA), heating the sample from 25 to 600 °C at a rate of 10 °C/min in a nitrogen atmosphere.

### 4.8. ORAC Antioxidant Assay

The antioxidant capacity of CSNFs was determined with an ORAC antioxidant assay kit (Abcam, Boston, MA, USA) according to the manufacturer’s instructions, as described by Dávalos et al. [115]. The reaction was carried out in a black 96-well flat-bottom read microplate (Abcam brand), using 75 mM phosphate buffer (pH 7.4) in a final reaction volume of 200 μL. The analysis was carried out with 25 µL of the nanoformulations, using ascorbic acid 50 μg/mL as a positive control (in triplicate). Then, 120 µL of fluorescein (70 mM) were added and the mixture was incubated at 37 °C for 15 min. After this period, 25 µL of the 2,2′-azobis(2-methylpropionamidine) daihydrochloride (AAPH) solution were added to initiate the reaction. The plate was placed in the Synergy H1M multi-mode microplate reader (Biotek, Santa Clara, CA, USA) and fluorescence was recorded every minute for 60 min (excitation: 458 nm; emission: 520 nm). Standard curves were generated for each sample using Trolox (Abcam, Boston, MA, USA) as antioxidant standard, ranging from 0 to 1000 μM (Figure S2). PBS buffer was used as blank instead of fluorescein. The area under the fluorescence decay curves (AUC) was determined for each sample by integrating the relative fluorescence curve (r^2^ > 0.99). ORAC values were expressed as μM of Trolox equivalents (μM TE) of CSNFs using the standard curve.

### 4.9. Cell Culture

The cells were cultured to 70–80% confluency in Petri plates in high glucose Dulbecco’s modified Eagle’s medium supplemented with 10% (*v*/*v*) fetal bovine serum, 100 U mL^−1^ penicillin and 100 μg mL^−1^ streptomycin. The cells were maintained in a humidified atmosphere of 5% CO_2_ and 95% air at 37 °C using an NB-203 incubator (N-BIOTEK, Bucheon, Republic of Korea). Once expanded, the cells were cultured in 96-well plates and in T75 cell culture flasks, depending on the assay to be performed. Growth medium was changed every 48 to 72 h and cells were viewed under an inverted microscope (AE2000, Motic, Xiamen, China) to estimate confluence. All experiments were conducted using HaCaT cells at passages 6–9.

### 4.10. In Vitro UVA Irradiation

The irradiation system consisted of a chamber equipped with six UV lamps emitting long-wave UVA radiation in the 350–400 nm range (Actinic BL-TL-K, Philips, Amsterdam, The Netherland). The irradiance of the lamps was 3 mW/cm^2^ at a distance of 10 cm from the cell culture plate, which was measured with an UV radiometer (CHY 732 UVA/Meter, Chy Firemate Co., Tainan, Taiwan, China). The incident dose of UVA received from above by the samples was calculated according to the equation below:

(3) $$UVA\;dosis\;\left(J/{cm}^{2}\right)=UVA\;irradiance\;(W/{cm}^{2})\cdot t(s)$$

The photoprotective effect of the MAA_RE_-loading CSNFs of was studied in HaCaT cells grown to a density of 10^4^ cells per well in a 96-well microplate. In the initial set of experiments, keratinocytes were treated with 3.4, 6.8 and 10.2 μg/mL of MAA-loaded CSNFs (from now on referred to as CSNFs-3.4, CSNFs-6.8 and CSNFs-10.2, respectively) and exposed to low–medium intensity UVA doses (5, 10, 15 and 30 J/cm^2^). In a more stringent experimental setting, cells were treated with 3.4 and 17 μg/mL of MAA-loaded CSNFs (from now on referred to as CSNFs-17) and subjected to a high-intensity UVA dose (60 J/cm^2^). Non-irradiated cells and non-treated cells were employed as controls to compare the photoprotective effects of the CSNFs. In addition, two approaches were investigated by irradiating the keratinocytes both before (pre-treatment) and after (post-treatment) incubation with the CSNFs: (i) Prior to UVA exposure, keratinocytes were grown to 80% confluence in 96-well culture plates, then washed with PBS buffer and incubated with 100 μL of CSNFs for 4 h at 37 °C; (ii) keratinocytes were cultured and treated following the same procedure, but in this case the cells were first exposed to UVA irradiation and subsequently incubated with the nanoformulations for 4 h at 37 °C. The culture medium was aspirated, and an equal volume (100 μL) of PBS was added to the cells before initiating UVA irradiation to maintain isovolumetric conditions. Following exposure, PBS was replaced with fresh medium, and the cells were kept for an additional period of 24 h before conducting viability and qPCR assays.

### 4.11. Cell Viability Assay

The effect of UVA irradiation on cell viability was evaluated colorimetrically in 96-well microplates using the XTT Cell Viability Assay Kit (Biotium, Fremont, CA, USA), which is based on the formation of a water-soluble orange formazan due to mitochondrial reduction of tetrazolium salt 2,3-bis[2-methyloxy-4-nitro-5-sulfophenyl]-2H-tetrazolium-5-carboxanilide (XTT). Briefly, 100 μL of cell culture medium was replaced by 100 µL of fresh medium with XTT working solution (final concentration 0.3 mg/mL) and PBS as a control. After incubation for 4 h at 37 °C, the absorbance of the plates was read between 400 and 450 nm with an Epoch^TM^ Microplate Spectrophotometer (Bio-Tek Instruments, Winooski, VT, USA).

### 4.12. RNA Extraction and PCR Assays

Total RNA was isolated from the HaCaT cells exposed to UVA irradiation using RNA-Solv (Omega Biotek, Norcross, GA, USA), according to the manufacturer’s instructions. The purity and concentration of the RNAs were quantified spectrophotometrically using a Epoch^TM^ Microplate Spectrophotometer (Bio-Tek Instruments, Winooski, VT, USA). The integrity of the isolated RNA was also checked by running it in 2% agarose gel electrophoresis. Total RNA (1 μg) was reverse transcribed to cDNA using Affinity Script cDNA Synthesis Kit (Agilent, Santa Clara, CA, USA) as per the instructions provided by the manufacturer.

PCR assay was performed in a thermal cycler QuantStudio^TM^ 3 System (Applied Biosystems, Foster City, CA, USA) using SapphireAmp Fast PCR Master Mix containing 0.5 μL of the sample DNA, 12.5 μL SapphireAmp^®^ Fast PCR Master Mix (Takara Bio, Shiga, Japan), 5 μL of each primer 1 μM (0.2 μM final concentration) and 2 μL nanopure water DNAase/RNAase free (25 μL final volume). PCR primers were purchased from Integrated DNA Technologies Inc. (IDT, Coralville, IA, USA), which amplify DNA fragments of (i) COX1 (cellular antioxidant response protein, cyclooxygenase; (ii) Keap1 (Kelch-like ECH-associated protein 1) and (iii) β-actin (housekeeping gene). Table 2 provides the details of the primer sequences and amplification products. The following thermocycling conditions were employed: 94 °C for 1 min, followed by 30 cycles of a 3-step program: 98 °C for 5 s, 55 °C for 5 s, 72 °C for 5 s and the samples were kept at 72 °C for 5 min. The PCR products were separated by electrophoresis in a 1% agarose gel and visualized by Red Gel staining with UV light illumination (MLB-16, Maestrogen, Hsinchu City, Taiwan). Gel images were acquired under UV illumination and analyzed using ImageJ software version 1.53k (NIH, Bethesda, MD, USA) following the previously described Full-Lane Quantification (FLQ) method [116]. Briefly, images were converted to 8-bit grayscale, then split into RGB channels and the red channel was selected for pixel intensity quantification. Band intensities were normalized to β-actin and expressed as fold change relative to untreated controls.

### 4.13. Statistical Analysis

Data shown are the mean ± standard error of at least three independent experiments. Statistical significance was determined at the 95% confidence level, using the non-parametric Mann–Whitney test to compare differences between two groups, and the non-parametric multiple ANOVA test for multiple comparisons.

## 5. Conclusions

The increasing incidence of UV-induced skin damage has led to the widespread use of chemical UV filters, many of which have raised concerns due to their potential risks to human health, the environment, and aquatic ecosystems. Herein, a novel CSNF incorporating an MAA-rich aqueous extract from *M. laminarioides* was developed and characterized, and its photoprotective efficacy was assessed in HaCaT human keratinocytes subjected to a broad UVA dose range (5–60 J/cm^2^). The CSNFs exhibited strong photoprotective efficacy in preserving cell viability under both pre- and post-treatment conditions, outperforming the effects reported in comparable state-of-the-art studies based on non-encapsulated extracts or purified algal MAAs. The protective effects of MAAs likely result from a combination of their UV-absorbing capacity, their ability to directly neutralize intracellular ROS via chemical quenching, and their well-documented role in activating molecular pathways associated with antioxidant defense and DNA repair mechanisms. In line with this, gene expression analysis accounted for a compensatory response, suggesting that CSNFs may stimulate prostaglandin-mediated cytoprotection. Therefore, our results further support the use of chitosan as an effective carrier for MAA-rich algal extracts to potentiate the photoprotective and bioactive efficacy of MAAs against UVA-induced damage. Overall, the combined evidence underscores the potential of CSNFs for the development of eco-friendly sunscreens based on a biopolymer and natural UV-absorbing compounds, offering a promising alternative to conventional synthetic chemicals thanks to their unique photophysical and biological properties.

## Figures and Tables

**Table 1:** Comparative analysis of photoprotective efficacy between the present study and previously reported MAA-based formulations.

Cell Line	MAAs	Formulation	MAAs Dose	UV Dose	Viability Without MAAs	Viability with MAAs	Photoprotective Efficacy (Fold Change in Viability)	Refs.
HaCat	Porphyra-334	HPLC-purified porphyra-334 in methanol 50% (*v*/*v*)	0.1 mg/mL	20 J/cm^2^, 300–400 nm (UVB/UVA)	39%	83%	2.2×	[22,24]
HaCaT (ATCC 12191)	MAA-rich algal extract of laver (*Porphyra yezoensis*)	80% (*v*/*v*) methanol extract of laver	0.5–3.0 mg/mL DW alga	70 mJ/cm^2^, (UVB)	68%	78% (with 3.0 mg/mL)	1.1× (post-treatment)	[51]
Human skin fibroblasts CCD-986sk	Porphyra-334	HPLC-purified porphyra-334 in 0.1% acetic acid (*v*/*v*)	0–40 µM (0–18.9 μg/mL)	10 J/cm^2^ (UVA)	68%	90% (with 18.9 μg/mL)	1.4×	[23]
HaCaT	Palythine	HPLC-purified palythine in PBS	0.3–10% *w*/*v* (3–100 mg/mL)	20 J/cm^2^ (UVA)	47%	100%	2.2×	[82]
HaCat	MAA-rich algal extract of luga cuchara (*Mazaella laminarioides*)	Nanoformulation	3.4–17 μg/mL	Low to medium UVA dose (5–30 J/cm^2^)	38%	92% (with CSNFs-10.2)	2.3–2.6× (pre-treatment)	This study
				High UVA dosis (60 J/cm^2^)	15%	62.5% (with CSNFs-17)	4.2× (pre-treatment) 8.5× (post-treatment)	

**Table 2:** Sequences specific to target genes and PCR fragment sizes.

Primer	Forward	Reverse	Fragment Size	Ref.
COX 1	5′-GGGCTTGGGCCATGGGGTAG-3′	5′-AGCTGCTCATCGCCCCAGGT-3′	318 pb	[117]
Keap 1	5′-CAGAGGTGGTGGTGTTGCTTAT-3′	5′-AGCTCGTTCATGATGCCAAAG-3′	244 pb	[118]
β-actin	5′-AGAGATGGCCACGGCTGCTT-3′	5′-ATTTGCGGTGGACGATGGAG-3′	406 pb	[118]

## Data Availability

The data presented in this study are available on request from the corresponding author.

## Supplementary Materials

The following supporting information can be downloaded at: https://www.mdpi.com/article/10.3390/ijms262110394/s1.

## Abbreviations

The following abbreviations are used in this manuscript:

MAAs	Mycosporine-like amino acids
UVR	ultraviolet radiation
CS	Chitosan
TPP	Sodium tripolyphosphate
CSNPs	Chitosan nanoparticles
ROS	Reactive oxygen species
