# Supplementary material for: Chitosan Nanoformulations of Mycosporine-like Amino Acid (MAA)-Rich Extracts from Mazzaella laminarioides Effectively Protect Human Keratinocytes Against UVA Radiation Damage
Source: Int J Mol Sci. 2025 Oct 25;26(21):10394. doi: 10.3390/ijms262110394 (PMC12609401; doi:10.3390/ijms262110394)
Supplement: Supplementary file 1 [file ijms-26-10394-s001.zip › ijms-3900206-supplementary.pdf]

Supplementary Materials

Chitosan Nanoformulations of Mycosporine-like Amino Acid (MAA)-Rich Extracts from *Mazzaella laminarioides* Effectively Protect Human Keratinocytes Against UVA Radiation Damage

Osmán Vásquez [1], Braulio Contreras-Trigo [1], Eileen Castillo [2], Neriel Contreras [1], Jessica Lemus [1], Felipe A. Zuniga [3], Karina Oyarce [2], Dariela Núñez [4], Víctor Díaz-García [1,*] and Patricio Oyarzún [1,*]

[1] Facultad de Ingeniería, Universidad San Sebastián, Concepción 4080871, Chile
[2] Facultad Ciencias, Universidad San Sebastián, Concepción 4080871, Chile
[3] Departamento de Bioquímica Clínica e Inmunología, Facultad de Farmacia, Universidad de Concepción, Concepción 4070386, Chile
[4] Facultad de Ciencias, Universidad Católica de la Santísima Concepción, Concepción 4090541, Chile
* Correspondence: victor.diazg@uss.cl (V.D.-G.); patricio.oyarzun@uss.cl (P.O.)

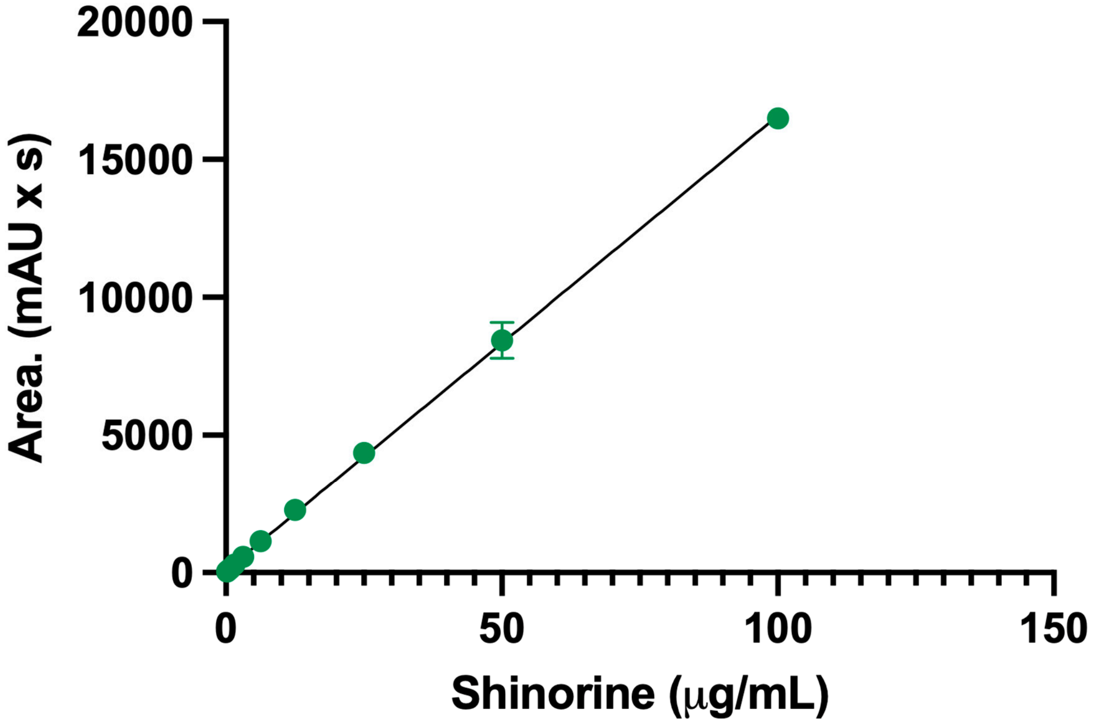

Figure S1. HPLC Calibration curve of standard shinorine

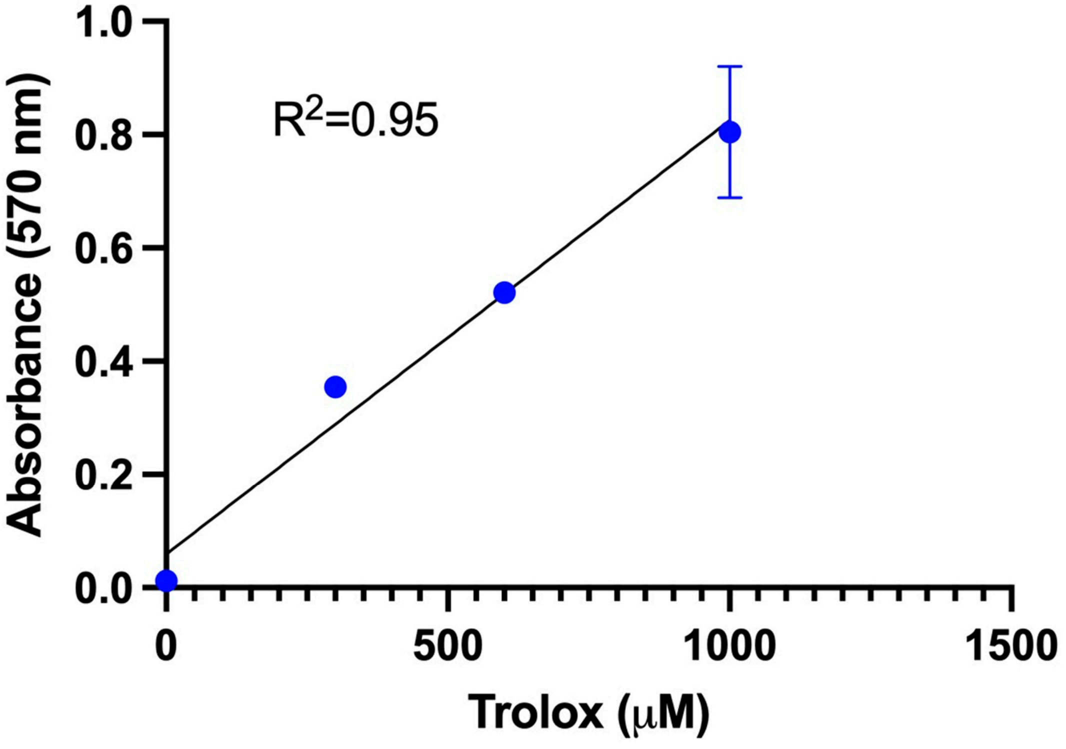

Figure S2. Trolox standard curve (ORAC assay)

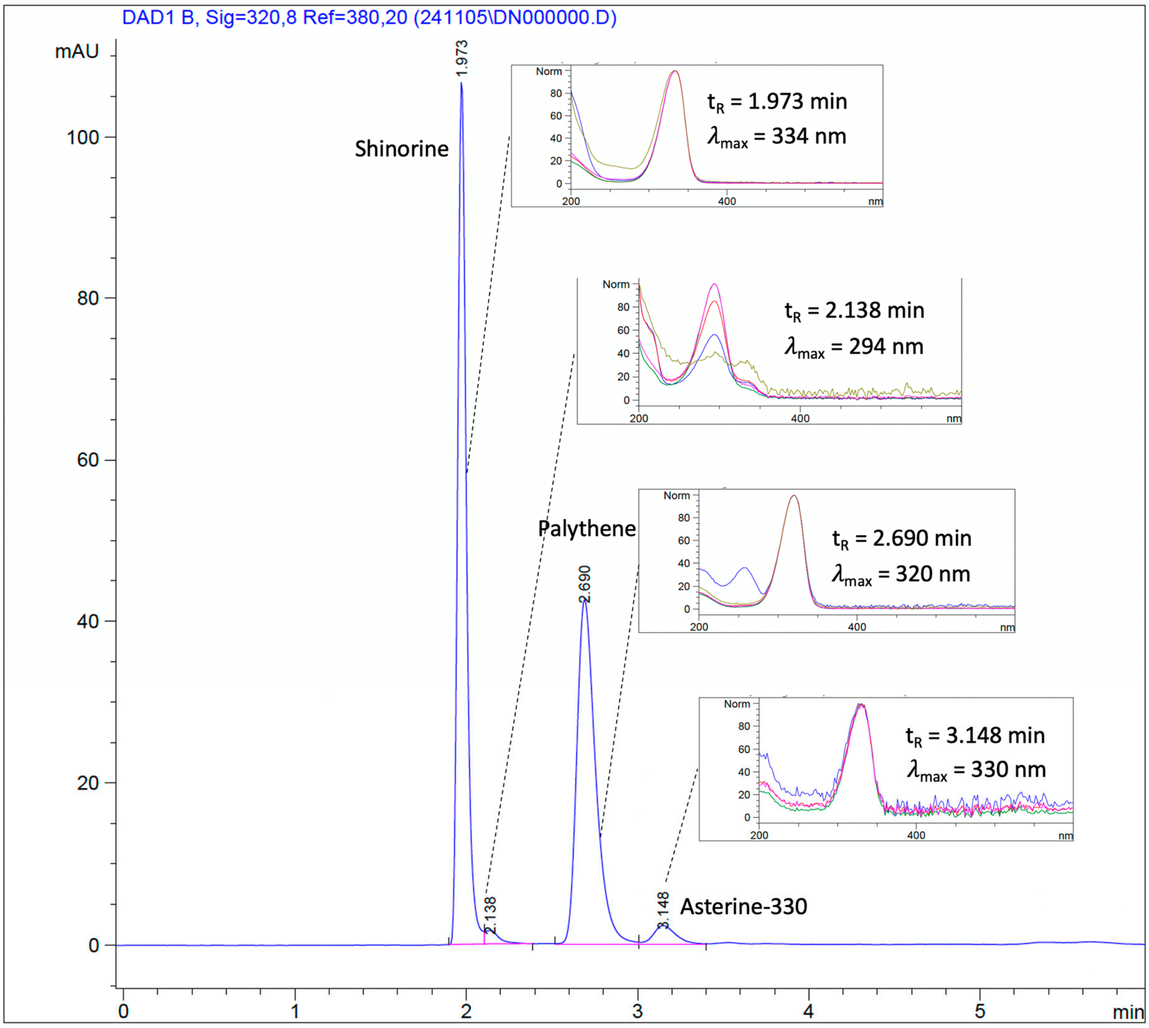

Figure S3. HPLC chromatogram of MAAs-rich algae extract, showing for each peak the characteristic retention time (t_R) and the wavelength of maximum absorbance (λ_{max}).

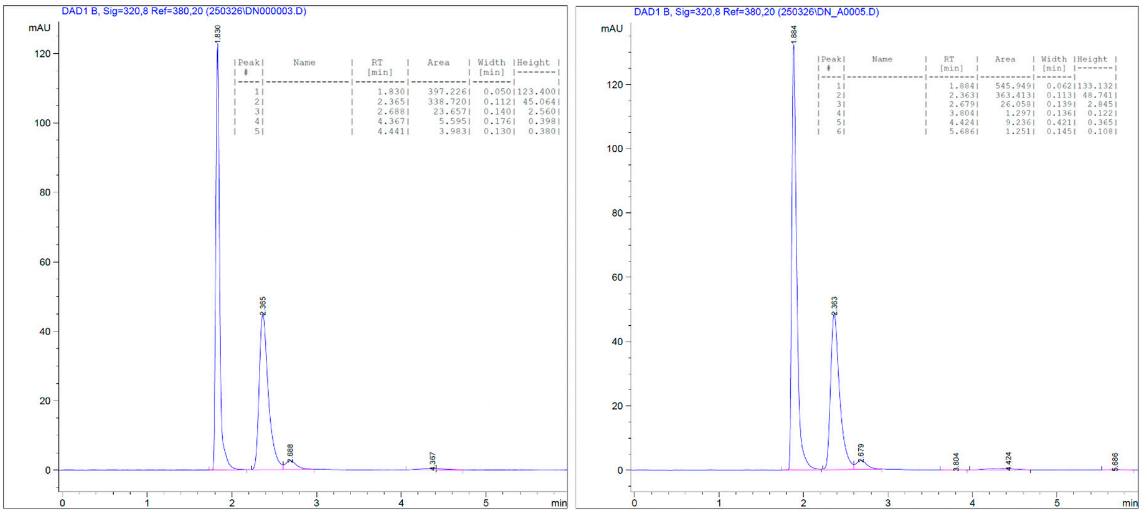

Figure S4. Chromatographic co-elution of the (A) MAAs-rich algae extract alone and (B) spiked with 1 μg/mL porphyra-334 standard.

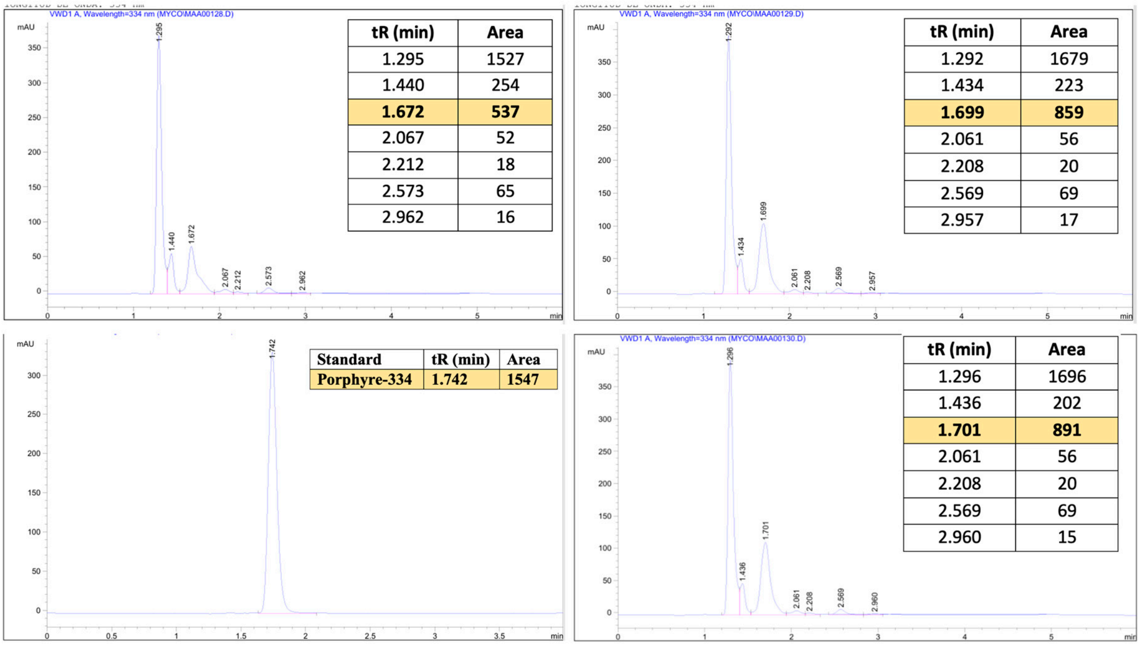

tR (min)	Area
1.295	1527
1.440	254
**1.672**	**537**
2.067	52
2.212	18
2.573	65
2.962	16

tR (min)	Area
1.292	1679
1.434	223
**1.699**	**859**
2.061	56
2.208	20
2.569	69
2.957	17

Standard	tR (min)	Area
**Porphyre-334**	**1.742**	**1547**

tR (min)	Area
1.296	1696
1.436	202
**1.701**	**891**
2.061	56
2.208	20
2.569	69
2.960	15

Figure S5. HPLC chromatograms of (A) MAAs-rich algae extract; (B) Pure porphyra-334 standard and (C and D) chromatographic co-elution of the algae extract with 1 μg/mL porphyra-334 standard (in duplicates)

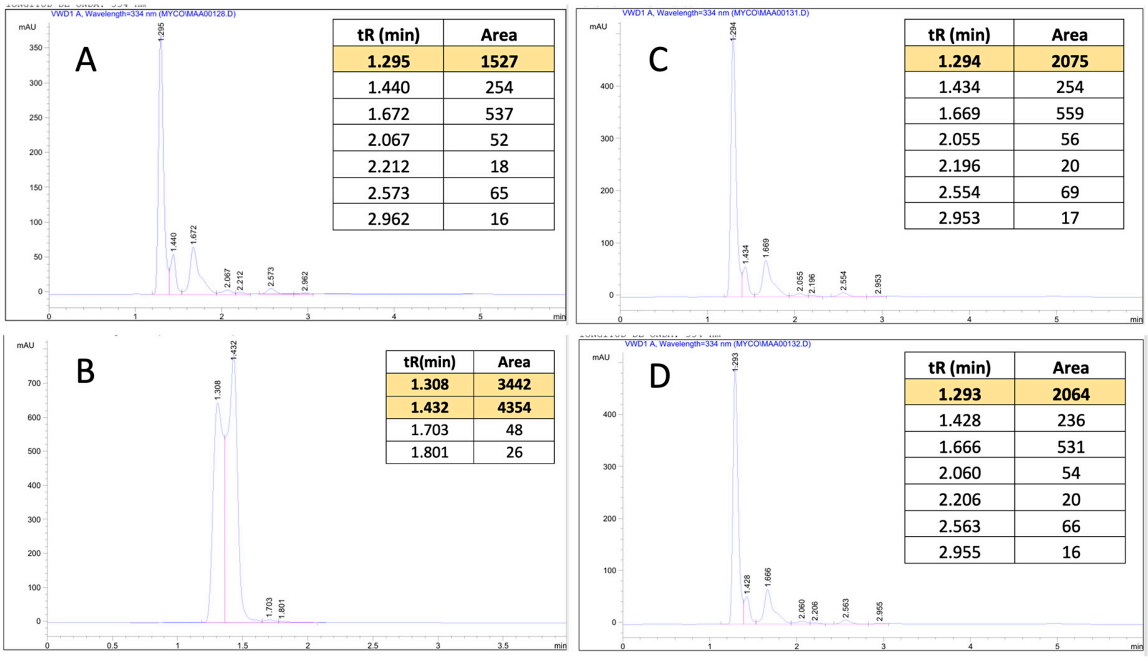

Figure S6. HPLC chromatograms of (A) MAAs-rich algae extract; (B) Pure shinorine standard and (C and D) chromatographic co-elution of the algae extract with 1 µg/mL shinorine standard (in duplicates)

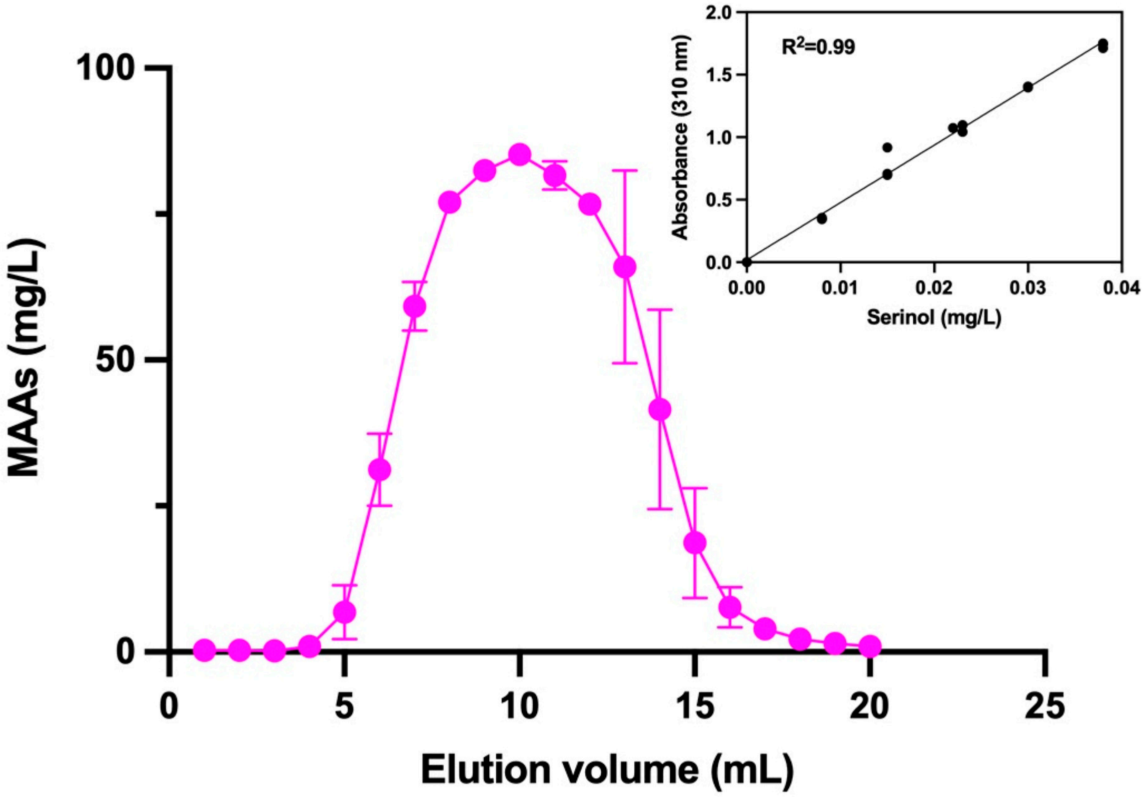

Figure S7. Elution profile of MAAs in the algae extract using a Sephadex G-10 column.

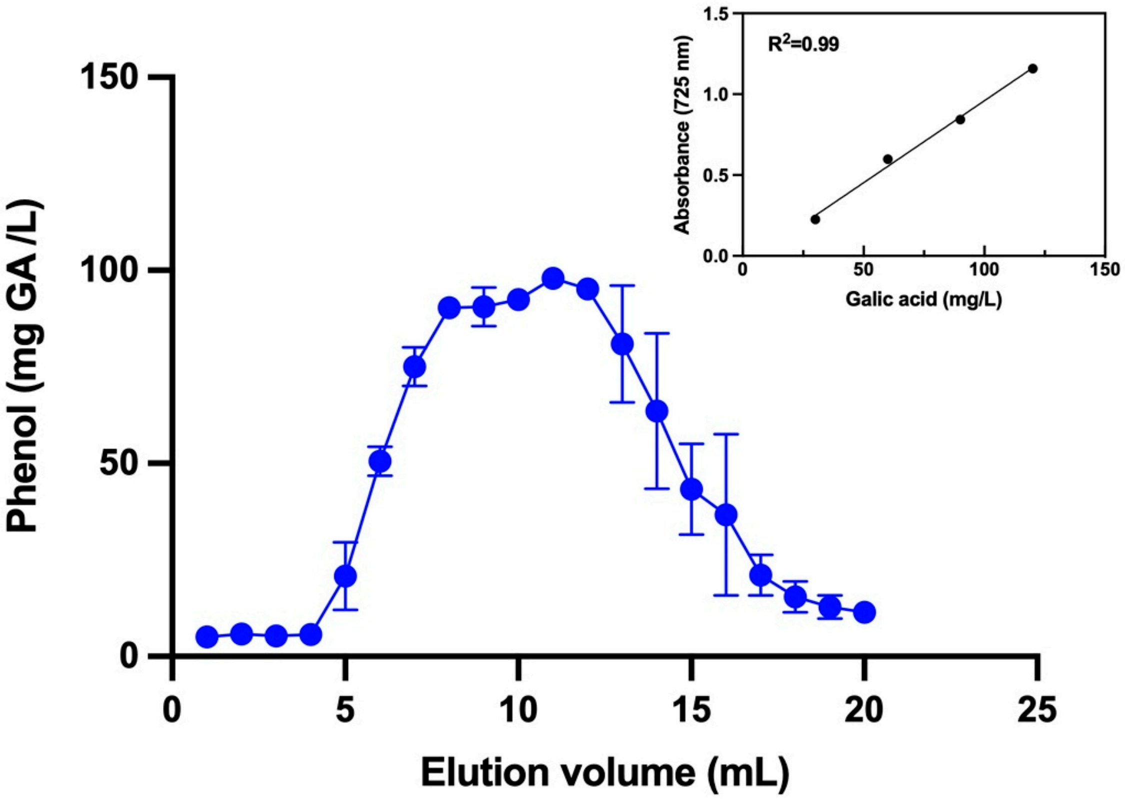

Figure S8. Elution profile of phenols in the algae extract using a Sephadex G-10 column.

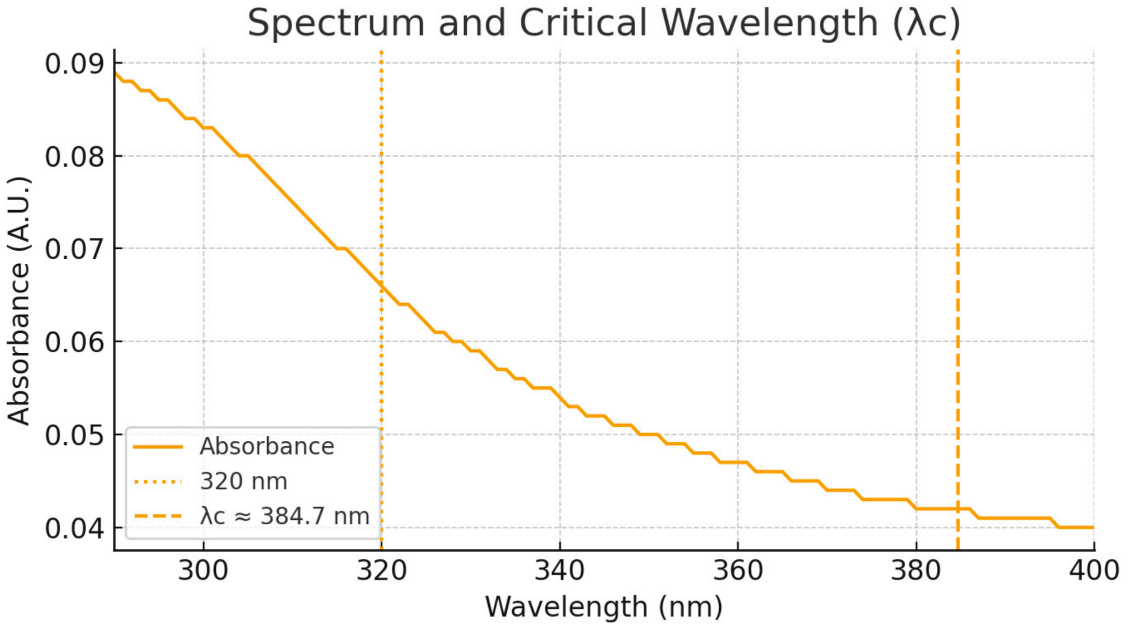

Figure S9. Determination of the critical wavelength from Integrated UVA/UVB Absorbance (290–400 nm) of the chitosan nanoformulation.

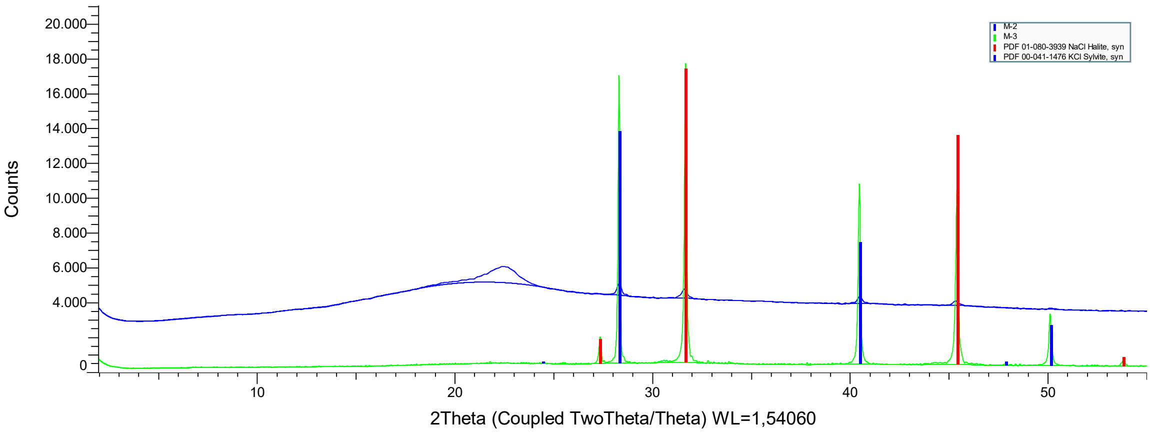

Figure S10. XRD patterns of CSNP and MAA raw extract.
